# Supplementary material for: Prevalence of multidrug resistance Salmonella species isolated from clinical specimens at University of Gondar comprehensive specialized hospital Northwest Ethiopia: A retrospective study
Source: PLoS One. 2024 May 7;19(5):e0301697. doi: 10.1371/journal.pone.0301697 (PMC11075907; doi:10.1371/journal.pone.0301697)
Supplement: Attachment — Submitted filename: PLoS one comments, questions and Recommendations.pdf [file pone.0301697.s003.pdf]

Reviewer’s comment, questions and Recommendations

Reviewer: Baye Gelaw Tarekegn (PhD; Professor of Medical Microbiology)

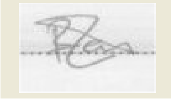

Full title of Manuscript: Prevalence of Multidrug Resistance Salmonella species isolated from clinical specimens at University of Gondar Comprehensive Specialized Hospital Northwest Ethiopia: A Retrospective study

Corresponding Author: Azanaw Amare

1. **General comments**

1.1: Strong points

- Study on drug susceptibility patterns of pathogens such as Salmonella species collected from clinical samples within six years period of time can be used as an evidence for designing strategies to combat the problem and that makes this study valuable.

- The main finding of the study was the prevalence of Salmonella Species which was 0.16%.

- Another important part of the study was the prevalence of drug resistance (As low as 16.7% and as high as 100%) and an overall MDR of Salmonella species which was 53.7%.

1.2: Limitations

- There were topographical, grammatical and punctuation error all over the entire document and I suggest that the manuscript requires language editorial by an expert.

- The study reports was extremely shallow although the data looks to some extent reasonable

2. **Detail Comments on each component of the manuscript**

2.1: Abstract

- The title and the abstract good and more or less convey what has been done but documented unsatisfactorily.

2.2: Introduction

- At the beginning of the introduction the authors documented the general characteristics, occurrence and pathogenesis of Salmonella species in two large paragraphs which is not adequate for such a manuscript rather was a general truth that doesn't correctly introduce the research title which is mainly about drug resistance and MDR. Moreover, no single evidence about the drug resistance pattern of these bacterial pathogens in Ethiopia and the Amhara National Regional state was introduced in this section.

- The authors must use quality English while organizing all the parts of the manuscript. In general, the literature review is not adequate and relevant research works that can demonstrate the magnitude of drug resistant salmonella species are not documented.

2.3: Methods

- At the end of the introduction part of the current manuscript, it was explained about the existence of the limited number of studies, lack of a coordinated epidemiological surveillance system, deficient reporting system, and lack of culture facilities in Ethiopia. It was also documented that understanding the prevalence and antimicrobial sensitivity pattern of Salmonella species will help to choose the right antimicrobial treatment and to reduce the spread of infection. However, the need to organize the retrospective data for publication was not adequately justified.

- The quality control methods used during data collection are not evidenced and don't guarantee the quality of the data.

- The objective of this retrospective study was described and well defined 'and' all the laboratory methods used were appropriate.

- In this study, the SOPs of the hospital laboratory were used but no new and specific protocols and algorithms particularly designed and applied in this study.

- The study was a retrospective study and data was generated from laboratory registration book.

- The only Software used in data analysis was SPSS version 26 and of course that does not disqualify the research work.

- Ethical approval was obtained from School of Biomedical and Laboratory Sciences but not evidenced with an official ethical clearance letter for which the issue number would have been documented. An evidence of the number, date and year of the issue of Letter of

permission from the University of Gondar Comprehensive Specialized Hospital would have been demonstrated.

2.4: Result

- At the beginning of the result section, Socio-demographic, prevalence and frequency of Salmonella species infection were presented and that is mixed. Why? The usual practice is demographic and clinical characteristics are separately documented and then compare that with the dependant variable.

- Please re-phrase line number 189 to 200.

- The prevalence of Salmonella species was 0.16% which is very low and what is the research finding and which question was answered by this result?

- Table one is extremity disorganized and the type of Salmonella bacteria proportion among patients from OPD, IPD and ICU was not known. In this table only age (Vertical column) was compared with other variable which is meaningless. Which one is the dependant variable for this study? Once the dependant variable identified, it should be used to evaluate the association with the independent variables. Table one is very deficient and it must be reorganized.

- The distribution of Salmonella species from different clinical samples was documented in table 2. What was the major aim of this part? So what is the scientific knowledge gathered from this table? It was reported by different investigators that Salmonella species can cause diseases in the GIT, UT, septicemia, wound infection and even meningitis.

- In this study, Data was presented in tables and proportions were described but Data was not analyzed properly. For example, there was no even a single evidence of association of the dependant variable with that of the independent variables.

- **Line number 16:** Trends of antibiotic resistance in Salmonella isolates: this part was interesting and the most important part of the manuscript but not analyzed properly. First, the authors must learn how to determine “trend” as a statistical term rather than comparison of the percentages. They can demonstrate the trend of AMR over time using graphs and determine if that is a statistically significant increases/decrease in AMR to the commonly used antibiotics. I advise the authors to discuss with

statisticians how to determine trend of the AMR and MDR Salmonelossis over the six years.

- In general, the results of the current study were not clearly presented and there is either very limited result or not at all that reflect the objectives of the study.

- In this study no new knowledge explained the study question except the patterns of drug resistance of the salmonella species.

2.5: Discussion

- The discussion shall begin with some findings about Salmonelosis Globally, in Africa and Ethiopia then followed by one of the finding of the current study but that was not done here. I strongly advice the authors to read, understand and look how to write discussion.

- Line number 33 to 35: Samonella infection versus age of the current study was compared with other studies and that was good. But, the way they are compared was not appropriate and the final reason presented on line number 35 and 36 is very immature. What does this discussion add on knowledge or practice in the pathogenesis, diagnosis, treatment and prevention of the disease? This kind of discussion is totally unacceptable. The authors must generate some reasons for the variation or demonstrate some of the reasons documented by literatures.

- Line number 37 to 43: This was also presented very poorly. What is the scientific evidence here for comparable, higher or lower prevalence of the Salmonella species at different study areas in Ethiopia and abroad? Otherwise, the discussion is meaningless!!

- Line number 43 to 48: most prevalent Salmonella species was *S. enterica* subsp. *arizonae* (51%) followed by *S. paratyphi* A (22%) and *S. typhi* 14.6%. This study is concordant with the study in Sagamu, Nigeria, but what was the result documented in Nigeria. Here, the reference was presented but there was no evidence for the similarity with the current study and that is totally unacceptable. Again I am forced to ask the authors to read how to organize a discussion and work again???

- Line number 53 to 61 looks good but need to be rephrased again!

- Line number 62 to 95: the antimicrobial resistance and MDR part would have been discussed better but this is a simple comparison with no convincing evidence or reason. Nothing was discussed about the factors that are highly valuable in drug resistance in Salmonellosis in general and in each of the species in particular.

- The limitation of the study may not be appropriate as the focus of the study was different from what was documented as limitations.

2.6: Conclusion and Recommendations

- The conclusion was good and it was based on the findings of the study but the recommendation was not based on the findings. For example one of the recommendation was stated as "Medical providers should use caution when administering antibiotics to patients with suspected of Salmonellosis during appropriate treatment" which is always true for any type of medication. That is not Unique to this outcome. Other recommendations are not also appropriate which means they are not study outcome based.

2.7: References

- There are references published as old as back to 1984, 2002 and 2008.

- References were organized using the Vancouver system?

2.8: Any additional comments?

With my capacity as reviewer, I would like to say the research question is good, but there are significant issues that need improvement and corrections in the abstract, introduction, method, result and discussion sections of the manuscript. **Therefore, re-analysis of data, and rigorous editing of the manuscript substantially needed. With this current status, my decision to the manuscript is that it needs major revision before accepting it for publication.**
