# Supplementary material for: Prevalence of multidrug resistance Salmonella species isolated from clinical specimens at University of Gondar comprehensive specialized hospital Northwest Ethiopia: A retrospective study
Source: PLoS One. 2024 May 7;19(5):e0301697. doi: 10.1371/journal.pone.0301697 (PMC11075907; doi:10.1371/journal.pone.0301697)
Supplement: Attachment — Submitted filename: Salmonella_University of Gondar comments.docx [file pone.0301697.s001.docx]

**Prevalence of Multidrug Resistance Salmonella species isolated from clinical specimens at University of Gondar Comprehensive Specialized Hospital Northwest Ethiopia: A Retrospective study**

Review comments

The authors have addressed a very important microbiological topic on antimicrobial resistance and pathogen profiles. Although it is retrospective, it offers enrichment to our current understanding of salmonellosis in the African context.

**Abstract:**

This is well written but requires editing to improve on the gramma.

**Introduction**

This chapter should be re-written.

Firstly, the statement *Genus Salmonella is the family Enterobacteriaceae which is a Gram-negative, non-lactose ferment* is not clear what exactly the author is trying to say.

Readers would be more interested in seeing the burden of salmonellosis across the globe, what is being done and the gaps. The authors should limit the characterization of the organisms at this stage as much of this is already documented elsewhere. Some grammatic errors also need to be corrected including the naming of drugs like naldixic acid, instead of nalidixic acid. This should be corrected throughout the document.

**Methods and materials**

The author should give us a feel of the hospital capacity eg: bedding, how many patients are seen in OPD and IPD in a month. How many of these have symptoms related to salmonellosis. This will guide us on the generalizability of the findings in this context.

Study population, sample size, and sampling technique

The authors repeatedly describe 2017-2022 as 6 years. This should be revised.

**Quality control**

The authors should include the QC and QA aspects that were done during culture and AST in the samples that were previously analyzed. Also, give a clear description of the exact QC that was done within your data collection. It is not clear what was checked by data collectors and how.

Results

The authors could include a figure of a study profile that would be helpful here.

**Discussion**

In the first paragraph, the authors describe salmonella prevalence figures in males and females. Ideally, these should add up to 100% but this is not the case. This should be explained.

In the fourth paragraph, the author should mention the countries in the narrative where the rates are lower or higher than what was observed in this study.
