# Supplementary material for: Prevalence of multidrug resistance Salmonella species isolated from clinical specimens at University of Gondar comprehensive specialized hospital Northwest Ethiopia: A retrospective study
Source: PLoS One. 2024 May 7;19(5):e0301697. doi: 10.1371/journal.pone.0301697 (PMC11075907; doi:10.1371/journal.pone.0301697)
Supplement: Attachment — Submitted filename: Response to Reviewers letter.docx [file pone.0301697.s002.docx]

# Review PLOS One

**Response letter for manuscript (PONE-D-23-08664)**

**To Academic** Editors and **reviewers:**

Dear Academic Editor and reviewers,

**Authors:** We the authors would like to thank the editorial teams of PLOS ONE for quick review process of our manuscript titled “***Prevalence of Multidrug Resistance Salmonella species isolated from clinical specimens at University of Gondar Comprehensive Specialized Hospital Northwest Ethiopia***”. We appreciate for spending your precious time and forwarding your valuable comments, which have significantly improved our application. We are also grateful for this positive feedback. Please see below, **bold,** for a point-by-point response to the reviewers. All page numbers refer to the revised manuscript file with tracked changes. All modifications in the manuscript have been highlighted in red.

We are looking forward to hearing from you in due course,

Sincerely,

Azanaw Amare (Corresponding author)
Responses to Editor’s comments

**Abstract:**

**Editor:** Make sure that the timeline for the research is reported consistently. It reads June 2017 to June 2017 in some lines. In addition, the abstract must show the methods used to identify Salmonella to the species level and the methods applied for the antimicrobial sensitivity measurements and interpretations. Further, you must clarify how the MDR was determined (one line in the abstract).

**Response: Thank you for this suggestion. It would have been interesting to explore this aspect. We tried to add the following suggestion on (page 2, Line: 38-43).**

**Introduction:**

Editor**:** The introduction is well documented, but it lacks a focus on Ethiopia. Other studies have explored the prevalence of Salmonella spp. in clinical settings in Ethiopia and AMR. The authors should briefly mention what is already known and how the current research enhances the topic's understanding (contribute insights).

**Response: We agree with this and have incorporated your suggestions and comments on (page 5, line: 97-104 and page 6, line: 120-125).**

**Methods:**

Editor: The main concern is identifying Salmonella at the species level, relying solely on a few biochemical tests. While biochemical systems such as API 20E could be used to identify Salmonella at the species level, the methods of choice are molecular. The authors should explain how they determined the species using only urease, TSI, indole motility, and lysine decarboxylase. Could the authors provide the final biochemical profiles for each identified species?

**Response: We agree with this and have incorporated your comments on (page 8, line: 176 -183). However molecular methods were taken as limitations (page 19, Line: 370–372).**

Editor**:** The Salmonella were retrieved from fecal, urine, or CSF samples. It’s unclear if the samples were from the same or different patients. The authors must clarify this.

**Response: We are also grateful for the suggestion and thank you. The Salmonella were retrieved either from fecal, urine, or CSF samples from different patients.**

Editor: For antimicrobial susceptibility testing, what were the quality controls in place? How were the intermediates isolates interpreted? Or were there no intermediates isolates?

**Response: Thank you for bringing this issue to our intention. We incorporate quality controls for antimicrobial susceptibility testing on (page 10, line: 208-214). However we did not found intermediate results during data collection**.

Editor Ethical consideration: Could the authors provide the ethics approval letter or unique identifier for the IRB approval?

**Response: Thank you for your comment. We have incorporated unique identifier for the ethical approval on (page 10, line: 223).**

Editor: The results make it unclear how many samples were sent to the microbiological laboratory and how many were Salmonella-suspected. Moreover, it’s unclear how the identified isolates were distributed in 2017-2022.

**Response: thank you for your direction. We tried to clarify using text and tables on (page 14)**

Editor: Could the authors make a table showing the demographics of the patients? The table should show the age, gender, region, and clinical symptoms of the patients (as this could support an understanding of the low detection level of Salmonella). Combining the demographics with the isolation/identification results makes reading difficult.

**Response: thank you for your direction. We tried to clarify the demographics of the patients (age, gender, and region) using tables on (page 12).**

Editor: Authors should revise the table titles by making them brief and descriptive.

**Response: Thank you for reflecting on your concern. We amended and made the table titles brief and descriptive through the manuscript.**

Editor: The discussion could be improved. The authors attempted to compare their findings to similar studies, but the interpretation of discrepancies is lacking. The authors often justify the discrepancies in methods used, region, etc. The authors should, for example, go further and explain why the methods used in this study (or the other compared studies) are not optimal for identifying Salmonella.

**Response: Thank you for your input. We tried to address all your concerns in the revised manuscript on (page 17: line 296-298, line 303-305, and line 309-311, page 18:line 314-316, line 325-327, and line 332-334 and page 19:line 344-348 and line 355-39) .**

Editor: There are many limitations to the current study. The significant limitations which reduce the reliability of the findings are:

1. Methods used for speciation of Salmonella.
2. Antibiotic Sensitivity testing that lacked standardization and quality control

**Response: Thank you for pointing out these potential limitations. We included this information in the** **limitation part on (page 19**, **Line: 370–372).**

Editor: Datasets must be available as per PLOS ONE policy.

**Response: authors to make all data necessary publicly available without restriction at the time of publication.**

**Response: We believed that we are following PLOS ONE’s style requirements**

Editor**:** There are many typos which reduce the quality of the manuscript. It’s recommended to proofread the manuscript before resubmission.

**Response: Thank you for your input. We attempted to proofread review the entire manuscript**

Please include the following items when submitting your revised manuscript:

- A rebuttal letter that responds to each point raised by the academic editor and reviewer(s). You should upload this letter as a separate file labeled 'Response to Reviewers'.

**Response: Thank you. We prepare and upload a response letter for each editor and reviewer(s) comments**

- A marked-up copy of your manuscript that highlights changes made to the original version. You should upload this as a separate file labeled 'Revised Manuscript with Track Changes'.

**Response: We prepared a track change file that highlights changes that we made to the original version during revision**.

- An unmarked version of your revised paper without tracked changes. You should upload this as a separate file labeled 'Manuscript'.

**Response: We prepared a revised manuscript without truck change and uploaded as 'Manuscript' in the submission system**

If you would like to make changes to your financial disclosure, please include your updated statement in your cover letter.
**Response: Thank you for your input. We updated the financial disclosure in the cover letter**.
If applicable, we recommend that you deposit your laboratory protocols in [protocols.io](http://protocols.io/) to enhance the reproducibility of your results. Protocols.io assigns your protocol its own identifier (DOI) so that it can be cited independently in the future. For instructions see: <https://journals.plos.org/plosone/s/submission-guidelines#loc-laboratory-protocols>. Additionally, PLOS ONE offers an option for publishing peer-reviewed Lab Protocol articles, which describe protocols hosted on [protocols.io](http://protocols.io/). Read more information on sharing protocols at <https://plos.org/protocols?utm_medium=editorial-email&utm_source=authorletters&utm_campaign=protocols>

**Response: NA**

Journal Requirements:

When submitting your revision, we need you to address these additional requirements.

Comments: 1. please ensure that your manuscript meets PLOS ONE's style requirements, including those for file naming. The PLOS ONE style templates can be found at

<https://journals.plos.org/plosone/s/file?id=wjVg/PLOSOne_formatting_sample_main_body.pdf> and

<https://journals.plos.org/plosone/s/file?id=ba62/PLOSOne_formatting_sample_title_authors_affiliations.pdf>.

**Response: During revision of our manuscript, we tried to follow the requirements of the journal PLOS ONE's style**

Comments: 2. please provide additional details regarding participant consent. In the ethics statement in the Methods and online submission information, please ensure that you have specified what type you obtained (for instance, written or verbal, and if verbal, how it was documented and witnessed). If your study included minors, state whether you obtained consent from parents or guardians. If the need for consent was waived by the ethics committee, please include this information.

**Response: Thank you for your comment. The participant consent was waived (obtained, documented and witnessed) by the ethics committee before we accessed the data. Please see it on page 10: line 223-227.**

Comments: 3. we note that you have stated that you will provide repository information for your data at acceptance. Should your manuscript be accepted for publication, we will hold it until you provide the relevant accession numbers or DOIs necessary to access your data. If you wish to make changes to your Data Availability statement, please describe these changes in your cover letter and we will update your Data Availability statement to reflect the information you provide.

**Response: Authors: Thank you. Please update it**

Comments: 4. please include a caption for figure 1.

**Response: Thank you for your input. We include the figure in caption.**

## **Responses to reviewers' comments**

Reviewer #1: Review comments
 Comments: the authors have addressed a very important microbiological topic on antimicrobial resistance and pathogen profiles. Although it is retrospective, it offers enrichment to our current understanding of salmonellosis in the African context.

**Response: We appreciate you taking the time to thoroughly review this manuscript and provide us with constructive feedback, which we believe improved the overall quality of our paper. We made every effort to address all your concerns. We've copied your comments and responses below to make things easier for you.**

Abstract:
Comments: This is well written but requires editing to improve on the gramma.

**Response: We are grateful for the suggestion and thank you. We went throughout the entire manuscript to eliminate grammatical and editing mistakes**.
Introduction
**Comments**: This chapter should be re-written.
Firstly, the statement Genus Salmonella is the family Enterobacteriaceae which is a Gram-negative, non-lactose ferment is not clear what exactly the author is trying to say.
Readers would be more interested in seeing the burden of salmonellosis across the globe, what is being done and the gaps. The authors should limit the characterization of the organisms at this stage as much of this is already documented elsewhere.

**Response: thank you very much for your direction, suggestion, and comments. We agree with this and have incorporated your suggestion in the revised manuscript on (page 3:line 64-65 and page 4: line 69-77).**

Comments: Some grammatic errors also need to be corrected including the naming of drugs like naldixic acid, instead of nalidixic acid. This should be corrected throughout the document.

**Response: Response: thank you very much for your direction, suggestion, and comments. We went throughout the entire manuscript to eliminate grammatical and editing mistakes**.

Methods and materials
Comments: The author should give us a feel of the hospital capacity eg: bedding, how many patients are seen in OPD and IPD in a month. How many of these have symptoms related to salmonellosis. This will guide us on the generalizability of the findings in this context.
**Response: Thank you for this suggestion. It would have been interesting to explore this aspect. We tried to add the following suggestion on (page 7: Line 140-145).
Comments**: Study population, sample size, and sampling technique
the authors repeatedly describe 2017-2022 as 6 years. This should be revised.
**Response: Thanks for your kind reminders. We revised it accordingly**.
Quality control
**Comments**: The authors should include the QC and QA aspects that were done during culture and AST in the samples that were previously analyzed. Also, give a clear description of the exact QC that was done within your data collection. It is not clear what was checked by data collectors and how.
**Response: thank you very much for your suggestions; the data collector were checked QC and QA in in records of QC and QA that was done by Laboratory technologist and we incorporate on (page 10 :line 208-214)**
Results
**Comments**: The authors could include a figure of a study profile that would be helpful here.
**Response: Thank you for your valuable suggestion and we incorporate in figure 1** Discussion
**Comments**: In the first paragraph, the authors describe salmonella prevalence figures in males and females. Ideally, these should add up to 100% but this is not the case. This should be explained.
**Response: Thank you for this suggestion. We went throughout the entire manuscript to eliminate editing mistakes**.
**Comments**: In the fourth paragraph, the author should mention the countries in the narrative where the rates are lower or higher than what was observed in this study.

**Response: Thank you for your input. We tried to address all your concerns in the revised manuscript page 18: line 324 – 325).**

 While revising your submission, please upload your figure files to the Preflight Analysis and Conversion Engine (PACE) digital diagnostic tool, <https://pacev2.apexcovantage.com/>. PACE helps ensure that figures meet PLOS requirements. To use PACE, you must first register as a user. Registration is free. Then, login and navigate to the UPLOAD tab, where you will find detailed instructions on how to use the tool. If you encounter any issues or have any questions when using PACE, please email PLOS at [figures@plos.org](mailto:figures@plos.org). Please note that Supporting Information files do not need this step.

**Response: Thank you for this suggestion. We have used PACE tool to increase the resolution of the figure**

**Authors**: **Finally, we would like to say thank you for reviewing our work and making insightful suggestions and comments that helped to strengthen our manuscript. We revised the manuscript as necessary**
