# Supplementary material for: Prevalence of multidrug resistance Salmonella species isolated from clinical specimens at University of Gondar comprehensive specialized hospital Northwest Ethiopia: A retrospective study
Source: PLoS One. 2024 May 7;19(5):e0301697. doi: 10.1371/journal.pone.0301697 (PMC11075907; doi:10.1371/journal.pone.0301697)
Supplement: Attachment — Submitted filename: Revew of the resubmision.docx [file pone.0301697.s004.docx]

Comments on the re-submitted manuscript

Thank you for responding to the review comments. The reviewer feels that some of the comments have not been well responded to. :Please respond appropriately to the following concerns

While the authors have attempted to elucidate the biochemical profile of the isolates, the current description remains rather generic, lacking specific details for each identity. It is suggested that the authors incorporate a schematic diagram in the manuscript to enhance clarity and provide a more concrete depiction of the biochemical profile associated with each isolate.

The authors have noted the absence of intermediate patterns in the Kirby Bauer AST method used. It is recommended that they include a table in the manuscript presenting the cut-off concentrations for each isolate or group of isolates, thereby offering a more detailed and informative representation of the study findings.

The authors have presented figures related to the samples from which Salmonella was isolated. However, for enhanced clarity, it is suggested that they include a schematic diagram. Additionally, it would be beneficial if the authors could provide information on the total number of samples sent to the lab and specify the count of samples that tested positive for Salmonella. This would contribute to a more comprehensive understanding of the study's outcomes.

Certain table titles still contain errors, such as "at at." It is recommended that the authors carefully review and revise the tables to ensure accuracy and eliminate any such mistakes in the titles.
