# Supplementary material for: Prevalence of multidrug resistance Salmonella species isolated from clinical specimens at University of Gondar comprehensive specialized hospital Northwest Ethiopia: A retrospective study
Source: PLoS One. 2024 May 7;19(5):e0301697. doi: 10.1371/journal.pone.0301697 (PMC11075907; doi:10.1371/journal.pone.0301697)
Supplement: Attachment — Submitted filename: Response to Reviewers.docx [file pone.0301697.s005.docx]

# Review PLOS One

**Response letter for manuscript (PONE-D-23-08664R1)**

**To Academic** Editors and **reviewers:**

Dear Academic Editor and reviewers,

**Authors:** We the authors would like to thank the editorial teams of PLOS ONE for quick review process of our manuscript titled “***Prevalence of Multidrug Resistance Salmonella species isolated from clinical specimens at University of Gondar Comprehensive Specialized Hospital Northwest Ethiopia***”. We appreciate for spending your precious time and forwarding your valuable comments, which have significantly improved our application. We are also grateful for this positive feedback. Please see below, **bold,** for a point-by-point response to the reviewers. All page numbers refer to the revised manuscript file with tracked changes. All modifications in the manuscript have been highlighted in red.

We are looking forward to hearing from you in due course,

Sincerely,

Azanaw Amare (Corresponding author)

Responses to reviewers’ comments

Reviewer 1

Comments on the re-submitted manuscript

Thank you for responding to the review comments. The reviewer feels that some of the comments have not been well responded to: Please respond appropriately to the following concerns

Reviewer comment: While the authors have attempted to elucidate the biochemical profile of the isolates, the current description remains rather generic, lacking specific details for each identity. It is suggested that the authors incorporate a schematic diagram in the manuscript to enhance clarity and provide a more concrete depiction of the biochemical profile associated with each isolate.

**Response: thank you very much for your suggestions and direction. We have tried to illustrate schematic diagram of the biochemical profile of the isolates in figure 1.**

Reviewer comment: The authors have noted the absence of intermediate patterns in the Kirby Bauer AST method used. It is recommended that they include a table in the manuscript presenting the cut-off concentrations for each isolate or group of isolates, thereby offering a more detailed and informative representation of the study findings.

**Author response: thank you very much for your remainder.  We accept the comment. The isolates with intermediate results detected were very few in numbers, however, for analysis counted as resistant.**

Reviewer comment: The authors have presented figures related to the samples from which Salmonella was isolated. However, for enhanced clarity, it is suggested that they include a schematic diagram. Additionally, it would be beneficial if the authors could provide information on the total number of samples sent to the lab and specify the count of samples that tested positive for Salmonella. This would contribute to a more comprehensive understanding of the study's outcomes.

**Author response: thank you very much for your direction. We have included schematic diagram count of samples that tested positive for *Salmonella* species in figure 2.**

Reviewer comment: Certain table titles still contain errors, such as "at at." It is recommended that the authors carefully review and revise the tables to ensure accuracy and eliminate any such mistakes in the titles.

**Author response: thank very much for your critical view. We have revised such types typing errors throughout the manuscript.**

Reviewer 2

General comments
Reviewer comment:1.1: Strong points
➢ Study on drug susceptibility patterns of pathogens such as Salmonella species collected from
clinical samples within six years period of time can be used as an evidence for designing
strategies to combat the problem and that makes this study valuable.

➢ The main finding of the study was the prevalence of Salmonella Species which was 0.16%.: ➢ another important part of the study was the prevalence of drug resistance (As low as 16.7%
and as high as 100%) and an overall MDR of Salmonella species which was 53.7%.

**Author response: thank you very much for your positive feedback.**
1.2: Limitations
Reviewer comment: ➢ There were topographical, grammatical and punctuation error all over the entire document and I suggest that the manuscript requires language editorial by an expert.

**Author response:** **We are grateful for the suggestion and thank you. We went throughout the entire manuscript to eliminate grammatical and editing mistakes (The manuscript is edited by Solomon Getawa Reta, PhD candidate; at Ariel University).**
Reviewer comment: ➢ The study reports was extremely shallow although the data looks to some extent reasonable

**Author response: thank very much for your positive feedback**.
2. Detail Comments on each component of the manuscript
2.1: Abstract
Reviewer comment: ➢ The title and the abstract good and more or less convey what has been done but documented unsatisfactorily.

**Author response: thank very much for your nice comments. We have revised the abstract**
2.2: Introduction
Reviewer comment: ➢ At the beginning of the introduction the authors documented the general characteristics, occurrence and pathogenesis of Salmonella species in two large paragraphs which is not adequate for such a manuscript rather was a general truth that doesn’t correctly introduce the research title which is mainly about drug resistance and MDR. Moreover, no single
evidence about the drug resistance pattern of these bacterial pathogens in Ethiopia and
the Amhara National Regional state was introduced in this section.

**Author response: thank very much for your constructive comments. We have revised the introduction as reviewer suggests. (See in page 4&5, line: 92-110)**
Reviewer comment:➢ The authors must use quality English while organizing all the parts of the manuscript.

**Author response: We tried eliminating editorial problems, and punctuation errors thought out the revised manuscript**

Reviewer comment: In general, the literature review is not adequate and relevant research works that can demonstrate the magnitude of drug resistant salmonella species are not documented.

**Author response: We agree with this and have incorporated your comments on (page 5, line: 101-106).**
2.3: Methods
Reviewer comment:➢ At the end of the introduction part of the current manuscript, it was explained about the existence of the limited number of studies, lack of a coordinated epidemiological surveillance system, deficient reporting system, and lack of culture facilities in Ethiopia. It was also documented that understanding the prevalence and antimicrobial sensitivity
pattern of Salmonella species will help to choose the right antimicrobial treatment and to
reduce the spread of infection. However, the need to organize the retrospective data for
publication was not adequately justified.

**Author response: thank you very much for direction. We have revised as reviewer suggested. (See in page 5, line 107-110)**
Reviewer comment: ➢ The quality control methods used during data collection are not evidenced and don’t guarantee the quality of the data.

**Author response: thanks for your feedback. The quality control methods were evaluated during data collection from weekly and daily and weekly quality control record books.**
Reviewer comment: ➢ the objective of this retrospective study was described and well defined ‘and’ the entire laboratory methods used were appropriate.

**Author response: thank very much for your positive feedback.**
Reviewer comment: ➢ in this study, the SOPs of the hospital laboratory were used but no new and specific protocols and algorithms particularly designed and applied in this study.

**Author response: we were documented the SOPs of hospital laboratory designed for *Salmonella* species identification.**
Reviewer comment: ➢ the study was a retrospective study and data was generated from laboratory registration book.

**Author response: thank very much for your comments. We have taken the data from Medical Microbiology Laboratory registration book**.

Reviewer comment: ➢ the only Software used in data analysis was SPSS version 26 and of course that does not disqualify the research work.

**Author response: we have used SPSS version 2 Software to enter and analysis the frequency and factor.**
Reviewer comment: ➢ Ethical approval was obtained from School of Biomedical and Laboratory Sciences but not evidenced with an official ethical clearance letter for which the issue number would have been documented. An evidence of the number, date and year of the issue of Letter of permission from the University of Gondar Comprehensive Specialized Hospital would
have been demonstrated.

**Author response: thank you very much for your comments. We have stated the reference number, date and year.**

2.4: Result
Reviewer comment: ➢ At the beginning of the result section, Socio-demographic, prevalence and frequency of Salmonella species infection were presented and that is mixed. Why? The usual
practice is demographic and clinical characteristics are separately documented and
then compare that with the dependent variable.

**Author response: thank you for your nice suggestions. We have separately presented Socio-demographic, prevalence and frequency of *Salmonella* species infection.**
Reviewer comment: ➢ please re-phrase line number 189 to 200.

**Author response: thank very much for your direction. We have re-phrased it accordingly.**
Reviewer comment: ➢ the prevalence of Salmonella species was 0.16% which is very low and what is the research finding and which question was answered by this result?

**Author response: thank you for your important question. The findings of this study is answered the prevalence of Salmonella species in different clinical sample.**
Reviewer comment: ➢ Table one is extremity disorganized and the type of Salmonella bacteria proportion among patients from OPD, IPD and ICU was not known. In this table only age (Vertical column) was compared with other variable which is meaningless. Which one is the dependent variable for this study? Once the dependent variable identified, it should be used to evaluate the association with the independent variables. Table one is very deficient and it must be reorganized.

**Author response: thank you very much for your direction. We have reanalysis again and tried to show the association of dependent variable with independent variables (see in page 10 & 12).**
Reviewer comment: ➢ The distribution of Salmonella species from different clinical samples was documented in table 2. What was the major aim of this part? So what is the scientific knowledge gathered from this table? It was reported by different investigators that Salmonella species can cause diseases in the GIT, UT, septicemia, wound infection and even meningitis.

**Author response: thank very much for your nice question. The main aim of this table is to know the distribution of salmonella species in different clinical samples.**
Reviewer comment: ➢ In this study, Data was presented in tables and proportions were described but Data was not analyzed properly. For example, there was no even a single evidence of association of the dependent variable with that of the independent variables.

**Author response: thank you for your reminder. We are analyzed the association of independent variable and dependent variables (Seen in page 14)**
Reviewer comment: ➢ Trends of antibiotic resistance in Salmonella isolates: this part was
interesting and the most important part of the manuscript but not analyzed properly. First, the authors must learn how to determine “trend” as a statistical term rather than comparison of the percentages. They can demonstrate the trend of AMR over time using graphs and determine if that is a statistically significant increases/decrease in AMR to the commonly used antibiotics. I advise the authors to discuss with statisticians how to determine trend of the AMR and MDR Salmonelossis over the six years.

**Author response: thank very much your valuable comments. We have consulted the statisticians to analyses the trend trends of antibiotic resistance in Salmonella isolates and corrected as reviewer suggestion (see in page 16).**
Reviewer comment: ➢ In general, the results of the current study were not clearly presented and there is either very limited result or not at all that reflect the objectives of the study.

**Author response: thank you for your comments. We revised the manuscript to relate with the objective**.
Reviewer comment: ➢ In this study no new knowledge explained the study question except the patterns of drug resistance of the salmonella species.

**Author response: thank you very much for comments. We try to show the prevalence of and MDR *Salmonella* isolates.**
2.5: Discussion
Reviewer comment: ➢ The discussion shall begin with some findings about Salmonellosis Globally, in Africa and Ethiopia then followed by one of the finding of the current study but that was not done here. I strongly advice the authors to read, understand and look how to write discussion.

**Author response: Thank you for your input. We tried to address all your concerns in the revised manuscript on (page 17: line, 312-318)**
Reviewer comment: ➢ Line number 33 to 35: Salmonella infection versus age of the current study was compared with other studies and that was good. But, the way they are compared was not appropriate and the final reason presented on line number 35 and 36 is very immature. What does this discussion add on knowledge or practice in the pathogenesis, diagnosis, treatment and prevention of the disease? This kind of discussion is totally unacceptable. The authors must generate some reasons for the variation or demonstrate some of the reasons documented by literatures.

**Author response: thank you for your constrictive comments. We have revised it as reviewer indicated (see in page 17, line 321-324)**.
Reviewer comment: ➢ Line number 37 to 43: This was also presented very poorly. What is the scientific evidence here for comparable, higher or lower prevalence of the Salmonella species at different study areas in Ethiopia and abroad? Otherwise, the discussion is meaningless!!

**Author response: thank you very much for your nice view. We revised it as reviewer suggested ( see in page 17 line, 328-331)**
Reviewer comment: ➢ Line number 43 to 48: most prevalent Salmonella species was S. enterica subsp. arizonae (51%) followed by S. paratyphi A (22%) and S. typhi 14.6%. This study is concordant with the study in Sagamu, Nigeria, but what was the result documented in Nigeria. Here, the reference was presented but there was no evidence for the similarity with the current study and that is totally unacceptable. Again I am forced to ask the authors to read how to organize a discussion and work again???

**Author response: thank you for your critical comments. We have revised and removed it accordingly.**
Reviewer comment: ➢ Line number 53 to 61 looks good but need to be rephrased again!

**Author response: Thank you very much for reminder. We have rephrased it.**

Reviewer comment: ➢ Line number 62 to 95: the antimicrobial resistance and MDR part would have been discussed better but this is a simple comparison with no convincing evidence or reason. Nothing was discussed about the factors that are highly valuable in drug resistance in Salmonellosis in general and in each of the species in particular.

**Author response: thank very much for comments. We have corrected it as reviewer suggests.**
Reviewer comment: ➢ The limitation of the study may not be appropriate as the focus of the study was different from what was documented as limitations.

**Author response: thank you very much for your comments. We have revised the limitations part as the reviewer suggestions**.
2.6: Conclusion and Recommendations
Reviewer comment: ➢ The conclusion was good and it was based on the findings of the study but the recommendation was not based on the findings. For example one of the recommendation was stated as “Medical providers should use caution when administering antibiotics to patients with suspected of Salmonellosis during appropriate treatment” which is always true for any type of medication. That is not Unique to this outcome. Other recommendations are not also appropriate which means they are not study outcome based.

**Author response: thank very much for you great full suggestion. We have corrected the** conclusion and recommendations as reviewer suggested.
2.7: References
Reviewer comment: ➢ There are references published as old as back to 1984, 2002 and 2008.

**Author response: thank you very much. We have updated the reference section accordingly**.
Reviewer comment: ➢ References were organized using the Vancouver system?

**Author response: we organized the reference using Vancouver referencing style.**
Reviewer comment: 2.8: Any additional comments?
With my capacity as reviewer, I would like to say the research question is good, but there are
significant issues that need improvement and corrections in the abstract, introduction, method,
result and discussion sections of the manuscript. Therefore, re-analysis of data, and rigorous
editing of the manuscript substantially needed. With this current status, my decision to the
manuscript is that it needs major revision before accepting it for publication.

**Author response: thank very much for your nice comments. We tried to re-analysis the data and eliminate editorial problems, and punctuation errors thought out the revised manuscript (The manuscript is edited by Solomon Getawa Reta, PhD candidate; at Ariel University).**
